# Hypogonadism and Metabolic Syndrome in Nigerian Male Patients With Both Type 2 Diabetes and Hypertension

**DOI:** 10.5812/ijem.10749

**Published:** 2014-01-01

**Authors:** Oluyemi Akinloye, Bolutife Blessing Popoola, Mary Bolanle Ajadi, Joseph Gregory Uchechukwu, Dolapo Pius Oparinde

**Affiliations:** 1 Department of Chemical Pathology, Faculty of Basic Medical Sciences, College of Health Sciences, Ladoke University of Technology, Osogbo, Osun State, Nigeria

**Keywords:** Testosterone, Metabolic Syndrome, Diabetes, Hypertension, Dyslipidemia, Cholesterol

## Abstract

**Background:**

The association between testosterone level and the components of metabolic syndrome remains controversial. Relevant studies from Sub-Saharan Africa are few and incohesive.

**Objectives:**

The current study was designed to investigate the level of testosterone in patients with both diabetes and hypertension and the association of low testosterone with metabolic syndrome in these patients.

**Materials and Methods:**

In this prospective case-control study, 83 male subjects (49 newly diagnosed men with both diabetes and hypertension and 34 apparently healthy controls) were recruited from Ladoke Akintola University of Technology Teaching Hospital, Osogbo, Nigeria and University College Hospital Ibadan, Ibadan, Nigeria. Demographic, anthropometric and sexual characteristics were obtained using structured questionnaires and standard methods. Blood plasma glucose (BPG), total cholesterol (TC), triglycerides (TG), high-density lipoprotein-cholesterol (HDL-C) and low-density lipoprotein-cholesterol (LDL-C) were measured by conventional methods. Testosterone (T) was analyzed by enzyme immunoassay. Data obtained were statically analyzed with the SPSS 15.0 software, and results were expressed as mean ± SEM.

**Results:**

This study showed significantly lowered concentrations of testosterone (3.11 nm/L ± 0.34) and HDL (0.39 mmol/L ± 0.02), in addition to the expected increased concentrations of fasting plasma glucose (9.61 mmol/L ± 0.37) in the subjects compared to controls (*P* < 0.05). An inverse significant correlation was observed between the serum testosterone concentration and metabolic syndrome (BMI, r = -0.477; waist/Hip ratio, r = -0.376 and dyslipidemia, r = -0.364, *P* < 0.05). Also, the testosterone level decreased with increase in central obesity (*P* < 0.05).

**Conclusions:**

This study established a strong association between low serum testosterone and metabolic syndrome in subjects with both type 2 diabetes and hypertension. It may therefore be advisable to include routine measurement of the testosterone level in the management of patients presented with both diabetes and hypertension. Furthermore, these patients may benefit from testosterone replacement therapy.

## 1. Background

Metabolic syndrome (MetS) is the combination of medical disorders that increases the risk of developing diabetes and cardiovascular disease (1). According to the World Health Organization (WHO), the MetS is a cluster of metabolic abnormalities, including centrally distributed obesity, decreased high density lipoprotein – cholesterol (HDL-C), elevated triglycerides (TG), hypertension, and hyperglycemia (2). Reaven (1988) (3) and Ferrannin (2007) (4) proposed that insulin resistance represented a fundamental “disorder” associated with a set of metabolic abnormalities which not only increased the risk of type 2 diabetes, but also contributed to the development of cardiovascular disease (CVD) before the appearance of hyperglycemia. 

Recent studies have implicated testosterone deficiency as a possible complication in men with type II diabetes and may contribute to impaired performance, mood and libido (5, 6). Although, a direct association between the testosterone deficiency and cardiovascular risk remains controversial, (7, 8) there is evidence that the testosterone levels are inversely associated with insulin resistance, (9) a potent risk factor for both micro- and macro-vascular complications for diabetes (10). Aging is associated with a gradual decline in testosterone (T) levels in men (11). This decrease is accompanied by changes in body composition including increases in fat mass and decreases in lean body mass, dyslipidemia, insulin resistance, and glucose metabolism dysregulation (12). Epidemiological evidence has shown that sex hormones are related to fasting plasma glucose, blood pressure and dyslipidemia in men, but not in all studies. In aging males, low serum T and sex hormone binding globulin (SHBG) have been associated with MetS in both cross-sectional and longitudinal design studies (13). These studies established that the prevalence of MetS increases with age and is related to hypogonadism. Moreover, a similar study suggested that the testosterone levels were lower in men with type II diabetes compared to patients with type I diabetes (14). Also, low testosterone levels have been associated with an increased risk of CVD and stroke (15). Furthermore, men with low testosterone levels have been reported to have higher blood pressure (16, 17). In addition, central or abdominal obesity, as measured by waist circumference, which is a classical feature of the MetS, has been independently associated with reduced testosterone levels (18). Svartberg et al. (17) not only discovered this association in a large number of community dwelling men, but also found that an increasing waist circumference predicted low testosterone (19). Studies from Sub-Saharan Africa are still few and incohesive on this subject.

## 2. Objectives

Hypertension is a risk factor for the development of renal and peripheral vascular diseases. Predisposing factors range from obesity, stress and presence of other ailments as renal and endocrine disorders. The incidence of hypertension has been reported to be higher in males than females (20). The mechanisms responsible for the increase in blood pressure in the males are unknown, but androgens have been shown to have a potential role in both humans and animals. Men with low testosterone levels have been reported to have higher blood pressure (16, 19) and low testosterone levels correlated with the higher blood pressure (21). Low testosterone levels have also been associated with increased risk of CVD and stroke (15), and also with spermatogenic failure and, consequently, infertility (22). However, the association between testosterone level and the components of MetS remains controversial. While there are few studies investigating testosterone level in either diabetic or hypertensive patients, especially in the developed countries, to the best of our knowledge, none looked at the testosterone level in patients with both diabetes and hypertension in men from Nigeria. Despite the controversy on the possible association between low testosterone and the components of MetS, few experimental studies are available. Therefore, this study was designed to investigate the association between serum level of testosterone and the severity of MetS in Nigerian male patients with both type 2 diabetes and hypertension.

## 3. Patients and Methods

### 3.1. Patients

The subjects of this case-control prospective study were newly diagnosed patients with both diabetes and hypertension recruited from The Outpatient Clinic of the Department of Medicine, Ladoke Akintola University of Technology Teaching Hospital, Osogbo, Osun State, Nigeria and The University College Hospital, Ibadan, Nigeria. Estimating the population of Nigeria with diabetes as 1.5 million (23) and the male to female ratio of 1.4:1, (24) the minimum sample size was calculated 32 in each study group using the formula n = Z^2^pq/d^2^ (n = sample size, Z = standard normal deviate – usually set at 1.96, p = proportion in the target population estimated to have a particular characteristics, q = 1 – p (proportion in the target population not having the particular characteristics), d = degree of accuracy required, usually set at 0.05) with a 95% confidence interval and a power of 80%. A total of randomly selected 83 subjects consisting of 49 consented patients (males) and 34 age-matched apparently healthy controls were recruited from the same environment. Informed consent was obtained from each of the subjects and participants answered a standardized questionnaire about lifestyle pattern and health history. Subjects who did not meet the selection criteria, which included other chronic diseases, history of drugs that could affect MetS or refused consent, were excluded. The study, received the approval of the Ethical Committee of Ladoke Akintola Teaching Hospital, Osogbo, Nigeria.

### 3.2. Anthropometrical Measurements

Anthropometrical measurements were taken using standard apparatus. A digital scale (Seca, Hamburg, Germany) was used to measure body weight (BW) with an accuracy of ± 100 g. Subjects were weighed without shoes, in light clothing. Standing body height (BH) was measured without shoes to the nearest 0.5 cm with the use of a commercial stadiometer with the shoulders in relaxed position and arms hanging freely. Body mass index (BMI) was then calculated as BW in kilograms (kg) divided by the square of the BH in meter (m^2^). Waist was measured horizontally at the level just above the uppermost border of the iliac crest. The measurement was made during normal minimal respiration. Hip was measured as the maximum circumference over the buttocks. Waist to hip ratio (WHR) was then calculated as an indicator of central obesity.

To measure blood pressure, subjects were seated in a chair with their back supported and their arms bared and supported at heart level. The appropriate cuff size was used to ensure an accurate measurement. Measurements were taken using a mercury sphygmomanometer applied on the right arm of the participants. First and fifth Korotkoff sounds were recorded for systolic and diastolic readings respectively.

### 3.3. Sampling 

After an overnight fast of 12 hrs, venous blood was drawn from the antecubital vein using a sterile syringe and needle into fluoride oxalate (FO) bottle, ethylene diamine tetra acetic acid (EDTA) and plain bottles. The samples were spun with a bench centrifuge at 4000 Rpm for 10 min. Plasma and serum were separated into plain bottles and stored at -20˚C until analysis. 

### 3.4. Assay of Glucose and Lipids

Plasma glucose was determined by the oxidase peroxidase enzymatic method (25). Glucose level was measured after enzymatic oxidation in the presence of glucose oxidase. The peroxide formed reacts under catalysis of peroxidase with phenol and 4-aminophenazone (4-aminoantipyrine) to form red-violet quinoneimine dye as indicator. Plasma triglyceride and cholesterol levels were assayed using commercial kits (Randox Laboratories, Crumlin, The UK) using the modified enzymatic method of Buccolo et al. (26) and Allain et al. (27), respectively. Very low-density lipoprotein (VLDL) and LDL were precipitated by the addition of phosphotungstic acid and magnesium chloride. After centrifugation at 3000 *g *for 10 min, the clear supernatant, which contained the HDL fraction, was analyzed for cholesterol using a Randox commercial kit (Randox Laboratories, Crumlin, The UK). Low-density lipoprotein-cholesterol (LDL-C) was calculated using the formula of Friedwald et al (28).

### 3.5. Measurement of Serum Testosterone

The testosterone level was measured by the enzyme immunoassay techniques developed for the Special Program Research in Human Reproduction by the WHO (2). The testosterone in the sample equilibrates with a fixed amount of alkaline phosphatase labeled Testosterone (Testosterone Label) in binding to a limited amount of monoclonal anti-Testosterone antibody. An anti-mouse IgG antibody bound to a magnetic particle was used to separate the Testosterone/Testosterone Label-antibody complex from the unbound component by magnetic sedimentation and a double wash step. The magnetic particles were incubated with enzyme substrate solution for a fixed time and reaction ended by adding stop buffer. The amount of color produced was inversely proportional to the amount of total testosterone present in the sample. The total testosterone concentration was interpolated from a calibration curve.

### 3.6. Statistical Analysis

Data analysis was performed using the SPSS 15 for Windows Software Package (SPSS Inc., Chicago, IL). Data were expressed as mean ± standard error of mean (SEM). The student T test was used to compare the mean between test and control. The associations between variables were analyzed with the Pearson’s correlation coefficient. Statistical significance was defined as *P *< 0.05. 

## 4. Results

Table 1 shows the anthropometric and biochemical values in cases and controls. The mean value of SBP in the control group was 117.79 ± 1.20 mmHg, DBP 73.82 ± 1.19 mmHg, BMI 22.32 ± 0.49 and waist to hip ratio of 0.90 ± 0.01. These values were significantly different (*P* < 0.05) from the test group with mean SBP values of 153.3 ± 1.13 mmHg, DBP 94.9 ± 2.22 mmHg, BMI 28.9 ± 0.53 kg/m^2^ and waist hip ratio of 0.97 ± 0.08 cm.

**Table 1.** Comparison of Measured Parameters in Cases and Controls

Parameters	Controls (n = 34)^a^	Tests (n = 49) ^a^	Student t-test	P values
**Age, y**	59.7 ± 2.62	63.8 ± 1.54	1.443	0.153
**BP ^b^-Systolic, mm/Hg**	117.8 ± 1.21	153.3 ± 1.13	20.190	0.000
**BP-Diastolic, mm/Hg**	73.8 ± 1.19	94.9 ± 2.22	7.401	0.000
**Height, cm**	1.68 ± 0.13	1.62 ± 0.09	3.609	0.001
**Weight, kg**	63.9 ± 1.66	76.3 ± 1.59	5.312	0.000
**BMI, kg/m^2^**	22.3 ± 0.49	28.9 ± 0.53	8.831	0.000
**Waist Circumference, cm**	83.2 ± 1.54	103.6 ± 1.63	8.720	0.000
**Hip Circumference, cm**	91.1 ± 1.36	103.7 ± 1.23	6.758	0.000
**Waist/Hip Ratio**	0.90 ± 0.08	0.97 ± 0.08	5.692	0.000
**FPG, mmol/L**	4.65 ± 0.17	9.61 ± 0.37	0.612	0.000
**TC, mmol/L**	3.64 ± 0.19	4.46±0.10	4.138	0.000
**TG, mmol/L**	1.77 ± 0.12	2.50 ± 0.09	5.019	0.000
**HDL, mmol/L**	0.92 ± 0.09	0.39 ± 0.22	6.448	0.000
**LDL, mmol/L**	2.00 ± 0.21	2.96 ± 0.94	4.666	0.000
**T, nmol/L**	34.0 ± 3.93	3.11 ± 0.34	7.697	0.000

^a^ Values expressed as mean ± standard error of mean. Differences between group are expressed by using student t – test with significant [*P* values set at <0.05]

^b^ Abbreviations: BMI, body mass index; SP, systolic blood pressure; DP, diastolic blood pressure; FPG, fasting plasma glucose; TC, total cholesterol; TG, triglycerides; HDL, high-density lipoprotein; LDL, low-density lipoprotein; T, testosterone.

**Figure 1.**
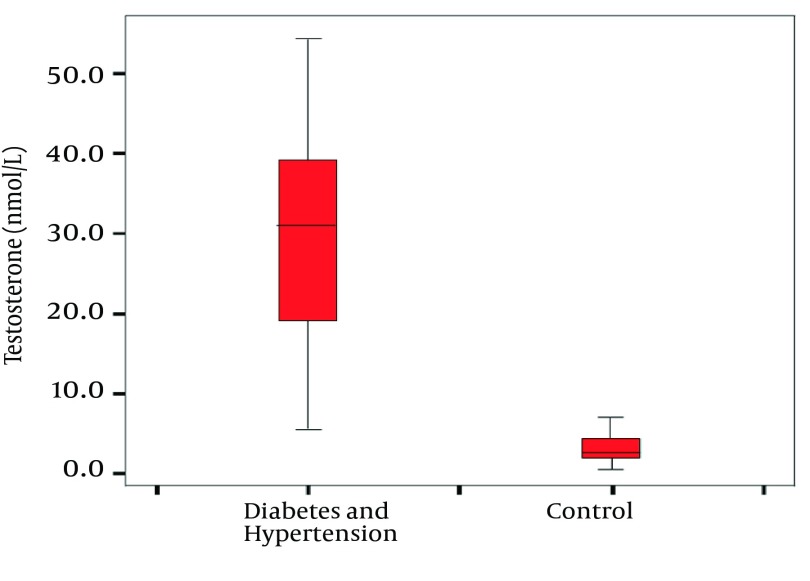
Box-Plots Showing the Median and Range of Testosterone Concentration in Patients and Controls

In our patients with both diabetes and hypertension, the mean value of fasting plasma glucose (9.61 ± 0.37 mmol/L) was very elevated, showing hyperglycemia as expected. The lipid profiles for TG, HDL-C, TC and LDL were 2.50 ± 0.09 mmol/L, 0.39 ± 0.02 mmol/L, 4.46 ± 0.10 mmol/L and 2.96 ± 0.94 mmol/L, respectively, showing significant dyslipidaemia. As shown in Figure 1, the serum testosterone level in patients (3.11 ± 0.34 nm/L) was significantly lower than in controls (34.3 ± 3.93 nm/L) (*P* < 0.05).

**Figure 2.**
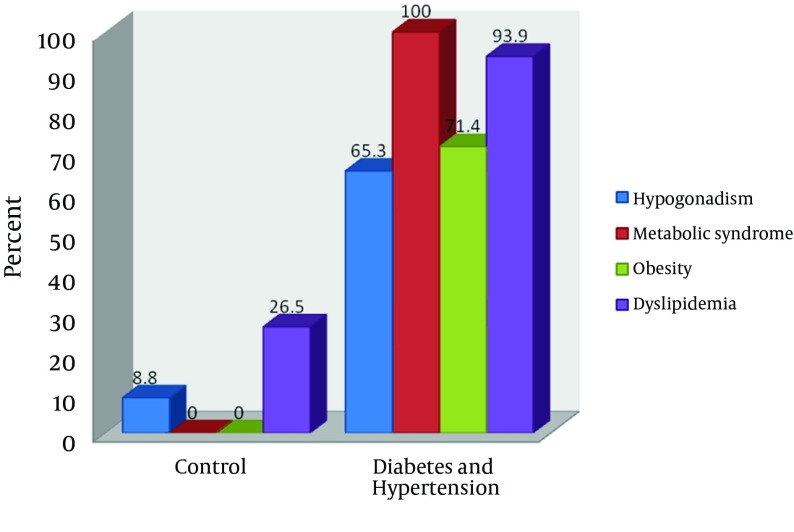
Distribution of Hypogonadism, Metabolic Syndrome, Obesity and Dyslipidemia for Patients and Controls

This significantly low serum testosterone correlated inversely with FPG, BP, BMI and WHR (*P* < 0.05). Table 2 shows a relevant correlation with the metabolic syndrome. Metabolic syndrome was the most common phenomenon in our investigated patients (100%) followed by dyslipidemia (93.9%), obesity (71.4%) and hypogonadism (65.3%), hence revealing a pattern of MetS > DYS > OBS > HYP, as illustrated in Figure 2. 

**Table 2.** Tailed Pearson Correlation of Investigated Parameters

	Age	SP ^b^	DP	BMI	W/H	FPG	TG	LDL	HDL	T2
**Age**	-	0.160	0.063	0.041	0.129	0.165	-0.125	-0.125	-0.130	-0.255^ a^
**SP **	0.160	-	0.605^a^	0.654^ a^	0.525^ a^	0.723^ a^	0.500^ a^	0.500^ a^	-0.545^ a^	-0.638
**DP**	0.063	0.605^ a^	-	0.523^ a^	0.385^ a^	0.457^ a^	0.288^ a^	0.288^ a^	-0.401^a^	-0.375^a^
**BMI**	0.041	0.654^ a^	0.523^ a^	-	0.556^ a^	0.558^ a^	0.456^ a^	0.456^ a^	-0.467^ a^	-0.477^ a^
**WHR**	0.129	0.525^ a^	0.385^ a^	0.556^ a^	-	0. 34^ a^	0.181	0.181	-0.331^ a^	-0.376^ a^
**FPG**	0.165	0.723^ a^	0.457^ a^	0.558^ a^	0.340^ a^	-	0.588^ a^	0.588^ a^	-0.416^ a^	-0.649^ a^
**TG**	0.125	0.500^ a^	0.288^ a^	0.465^ a^	0.181	0.588^ a^	-	0.462^ a^	0.364^ a^	-0.364^ a^
**LDL**	-0.125	0.500^ a^	0.288^ a^	0.456*	0.181	0.588^ a^	0.462^ a^	-	-0.416^ a^	0.085
**HDL**	-0.130	-0.545^ a^	-0.401^ a^	-0.467^ a^	-0.331^ a^	-0.461^ a^	-0.285^ a^	-0.416^ a^	-	0.250^ a^
**T2**	-0.255^ a^	-0.638^ a^	-0.375^ a^	-0.477^ a^	-0.376^ a^	-0.649*	0.364^ a^	0.085	0.250^ a^	-

^a^ Correlation is significant at 0.05 level (2-tailed)

^b^ Abbreviations: BMI, body mass index; SP, systolic blood pressure; DP, diastolic blood pressure; FPG, fasting plasma glucose; TC, total cholesterol; TG, triglycerides; HDL, high-density lipoprotein; LDL, low-density lipoprotein; T2, testosterone; WHR, waist to hip ratio.

**Figure 3.**
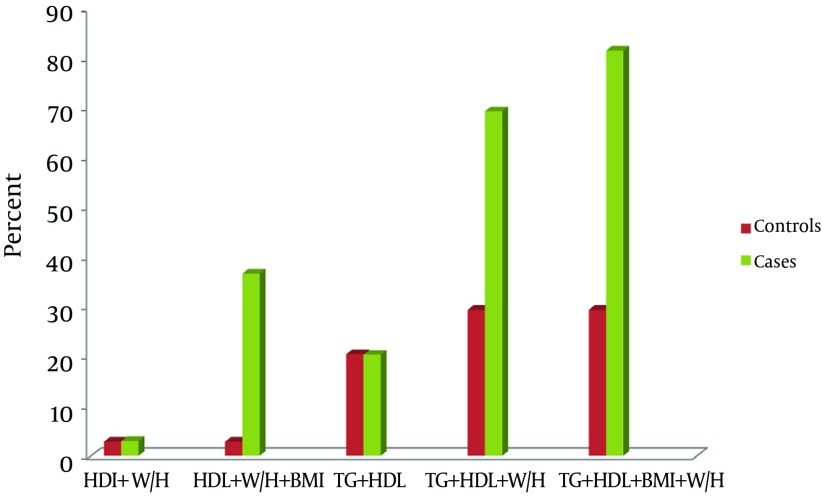
Cluster of Metabolic Syndrome Other Than Hyperglycemia and Hypertension BMI, body mass index; BP, blood pressure; FPG, fasting plasma glucose; TC, total cholesterol; TG, triglycerides; HDL, high-density lipoprotein; LDL, low-density lipoprotein; T2, testosterone; W/H, waist to hip ratio

However, when using three risk factors, the cluster of raised TG, reduced HDL and raised WHR, becomes the most promising marker of MetS in patients with diabetes and hypertension (replacing FBG and BP) as shown in Figure 3. 

## 5. Discussion

Epidemiological evidences have shown that sex hormones are related to fasting plasma glucose, blood pressure and dyslipidemia in men (13, 29). Recently, the association between hypogonadism and MetS has received more attention. This is because the prevalence of hypogonadism has been shown to be higher than previously thought in epidemiological studies (30). The current study is consistent with these reports. The serum testosterone in patients with both diabetes and hypertension is significantly lower than controls. Testosterone plays a critical role in male reproductive and metabolic functioning (31). Reduced testosterone or hypogonadism, as observed in this study, may be responsible for reduced libido, reduced reproductive performance and erectile dysfunction commonly reported in patients with diabetes. Studies have implicated testosterone deficiency as a possible complication in men with type II diabetes and implicated hypogonadism as a contributing factor to impaired performance, mood and libido in diabetes mellitus (5, 9). This is in agreement with our results with observed significant inverse correlation between low testosterone and fasting plasma glucose, indicating that the lower the testosterone the higher the fasting plasma glucose. A previous study has provided evidence that the testosterone levels are inversely associated with insulin resistance (9). Although a direct association between the testosterone deficiency and cardiovascular risk remains controversial, (7, 8) the inverse association with testosterone levels and insulin resistance, (9) a potent risk factor for both micro- and macro-vascular complications for diabetes as shown by Despres et al.(10) is a strong indication of its likely association with CVD. All the risk factors of cardiovascular diseases investigated in the current study, increased BMI (obesity), increased WHR (central obesity), reduced HDL and increased TG (dyslipidemia), showed a strong significant correlation with low testosterone (hypogonadism). This suggests that hypogonadism may play a more important role in the pathophysiology of micro- and macro-vascular complications commonly associated with diabetes and hypertension. Sixty five percent of the patients investigated in our study had hypogonadism. Although we could not establish if all the subjects with hypogonadism had sexual dysfunctions, it is clear from this study that hypogonadism is common and associated with diabetes and hypertension in Nigeria. Also, all investigated patients in this study (100%) had MetS, 93% dyslipidemia and 71.4% obesity, showing the possible contribution of these risk factors in diabetes and hypertension. The high percentage of dyslipidemia and obesity may be responsible for the absolute figure of MetS observed in this study. Svartberg et al. (17) suggested that waist circumference (WC) was superior to BMI in correlation with the components of MetS. This is in accordance with our study. Better still, we found the WHR to be superior to both factors. In fact, 69.4% of MetS patients were identified using WHR and dyslipidemia (high TG and low HDL) alone, compared to 36.7% when WHR was replaced with BMI. Svartberg et al. (19) reported that approximately 25% of obese individuals (BMI ≥ 30 kg/m²) had MetS. With other measures of adiposity, the maximum prevalence of MetS clustered was around 21%, suggesting that different measures of adiposity in the same study would yield different MetS prevalence values (17). This statement was confirmed by our study. However, the higher percentage using WHR observed in our study suggests that the cluster of TG, HDL and WHR are better indicators of MetS in Nigerian males. Furthermore, using both BMI and WHR in addition to dyslipidemia gave an increasing sensitivity (81.6%) in our study.

Corona et al. (32) reported that 96.5% of their subjects with MetS exhibited erectile dysfunction (ED) and of 154 men with organic ED, 43% had MetS, while the percentage of individuals expressing MetS increased with increasing the ED severity. The finding of hypogonadism in 65.3% of our subjects may explain the high ED associated with MetS. Men with MetS have been reported to have a higher risk of erectile dysfunction (ED) (33). Because MetS increases CV risk, it is not surprising that ED may also be a predictor of subsequent CVD. This is consistent with the evidence presented in the current study. Surprisingly, Paick et al. (34) did not find a significant association between ED severity and MetS parameters, except for hypertension in impotent men, suggesting that the association between MetS and ED severity may not be clear-cut, or may be selective for certain components. However, we found a strong inverse association, between low testosterone levels and MetS. This association further establishes the importance of low testosterone in reproductive dysfunction such as ED and the increase of MetS in our Nigerian subjects. The association between sex hormones and MetS has been reported to be statistically significant across racial/ethnic groups (35). This may explain the discrepancy in these studies. 

In conclusion, the current study in Nigerian males with diabetes and hypertension established a strong association between low testosterone levels and metabolic syndrome. This study then suggests that low testosterone level may be responsible for the reproductive dysfunction commonly associated with diabetes and hypertension. Furthermore, the study supports the previous reports, which included testosterone in the cluster for MetS diagnosis (36). Also, the study suggests that introducing WHR in the clustering of factors for MetS is a more sensitive indicator than BMI. This implies that the measurement of body fats may be a better indicator of CVD than BMI in patients with both diabetes and hypertension. Finally, it may be advisable to include routine measurements of the testosterone level in the management of patients presented with both diabetes and hypertension. The possible use of hormone (testosterone) replacement therapy to increase the life expectancy in the management of these patients needs to be elucidated.
